# Supplementary material for: Dietary Data in the Malmö Offspring Study–Reproducibility, Method Comparison and Validation against Objective Biomarkers
Source: Nutrients. 2021 May 9;13(5):1579. doi: 10.3390/nu13051579 (PMC8150333; doi:10.3390/nu13051579)
Supplement: Supplementary file 1 [file nutrients-13-01579-s001.zip › nutrients-1186160-supplementary.pdf]

Supplementary Material

Dietary Data in the Malmö Offspring Study – Reproducibility, Method Comparison and Validation against Objective Biomarkers

Sophie Hellstrand[1], Filip Ottosson[2], Einar Smith[2], Louise Brunkwall[1], Stina Ramne[3], Emily Sonestedt[3], Peter M. Nilsson[4], Olle Melander[2], Marju Orho-Melander[1] and Ulrika Ericson[1]

[1] Department of Clinical Sciences Malmö, Diabetes and Cardiovascular Disease-Genetic Epidemiology, Lund University, 205 02 Malmö, Sweden.
[2] Department of Clinical Sciences Malmö, Cardiovascular Research, Hypertension, Lund University, 205 02 Malmö, Sweden.
[3] Department of Clinical Sciences Malmö, Nutritional Epidemiology, Lund University, 205 02 Malmö, Sweden.
[4] Department of Internal Medicine, Skåne University Hospital, Lund University, 205 02 Malmö, Sweden.

CONTENTS

Supplementary Table 1. The 32 food items in the short food frequency questionnaire (SFFQ) used in the Malmö Offspring Study.
Supplementary Table 2. The metabolites identity confirmed by matching the plasma measurements of mass-over charge ratio and retention time with data acquired from the synthetic standards.
Supplementary Table 3. Mean and standard deviation for reported food intake from the first and second measurements; data from 4-d food records (4DFR) and the short food frequency questionnaire (SFFQ) in women and men from the Malmö Offspring Study.
Supplementary Table 4. Baseline daily nutrient intakes from the 4-d food record (4DFR) in the Malmö Offspring Study, and food intakes reported by the 4DFR that were not asked for in the short food frequency questionnaire (SFFQ) (*n*=1601).
Supplementary Table 5. Spearman correlations* between food intakes assessed by the 4-d food record (4DFR) (g/d) and the short food frequency questionnaire (SFFQ) (times/month and g/d for fish intake), in a subsample with repeated measurements 1.6 y later (*n*=180) in the Malmö Offspring Study.
Supplementary Table 6. Agreement between quartiles of intakes from specific fiber sources from the first and repeated 4-d food record (4DFR) in the Malmö Offspring Study (*n*=180).
Method explanation: Liquid chromatography-mass spectrometry.

Supplementary Table 1. *The 32 food items in the short food frequency questionnaire (SFFQ) used in the Malmö Offspring Study*

**Food item**
Low-fiber soft bread total
Low-fiber crispbread
High-fiber soft bread total
Medium high-fiber soft bread
Very high-fiber soft bread
High-fiber crisp bread
Fatty fish
Lean fish and shellfish
Fish products
Vegetables total
Legumes
Green leafy vegetables
Cruciferous vegetables
Onions
Tomatoes
Carrots
Other vegetables
Fruit and berries total
Fruits total
Citrus
Other fruits
Berries
Sugar-sweetened beverages
Low-calorie beverages
Energy/sport beverages
Butter for cooking
Margarine for cooking
Oil/liquid margarine for cooking
Oil/vinaigrette on salad
Energy bars/protein powder
Protein beverages
Food replacement products

Supplementary Table 2. *The metabolites identity confirmed by matching the plasma measurements of mass-over charge ratio and retention time with data acquired from the synthetic standards*

Metabolite	Chemical Formula	HMDB ID	m/z	Retention time
Proline betaine	$C_7H_{13}NO_2$	HMDB0004827	143.095	6.15
CMPF	$C_{12}H_{16}O_5$	HMDB0061112	239.092	9.80
β-carotene	$C_{40}H_{56}$	HMDB0000561	536.438	0.85

CMPF: 3-carboxy-4-methyl-5-propyl-2-furanpropanoic acid; HMDB ID: human metabolome database identification; m/z: mass-to-charge ratio.

Supplementary Table 3. *Mean and standard deviation for reported food intake from the first and second measurements; data from 4-d food records (4DFR) and the short food frequency questionnaire (SFFQ) in women and men from the Malmö Offspring Study*

	**1st 4DFR All women**	**1st 4DFR Subsample women**	**2nd 4DFR Subsample women**	**1st 4DFR All men**	**1st 4DFR Subsample men**	**2nd 4DFR Subsample men**
n	885	115	115	716	65	65
Low-fiber soft bread total (g/d)	52.9 (41.6)	57.6 (42.7)	50.8 (44.4)	82.6 (59.7)	75.5 (46.6)	64.7 (57.7)
High-fiber soft bread (g/d)	33.9 (44.5)	22.5 (28.5)	32.7 (48.8)	38.7 (50.4)	23.0 (29.0)	38.1 (28.1)
High-fiber crispbread (g/d)	6.9 (9.7)	8.0 (11.7)	7.5 (11.9)	5.1 (10.7)	4.0 (6.7)	3.7 (6.5)
Fatty fish (g/d)	15.1 (22.9)	18.9 (26.9)	14.1 (25.1)	17.5 (29.4)	21.6 (30.2)	20.0 (27.5)
Lean fish and shellfish (g/d)	17.1 (25.7)	18.5 (31.3)	17.3 (23.1)	16.1 (29.3)	14.8 (21.3)	15.7 (23.4)
Fish total (g/d)	32.2 (35.0)	37.4 (39.7)	31.4 (32.6)	33.6 (41.5))	36.4 (35.4)	35.6 (36.2)
Vegetables total (g/d)	176.0 (101.9)	190.5 (105.8)	198.7 (111.8)	171.5 (106.1)	148.5 (98.0)	166.3 (90.6)
Legumes (g/d)	11.4 (21.3)	13.5 (22.1)	18.1 (28.6)	11.8 (26.8)	9.7 (26.7)	11.6 (23.2)
Green leafy vegetables (g/d)	11.3 (15.1)	9.7 (11.2)	12.3 (13.2)	9.8 (14.3)	8.7 (10.9)	8.3 (9.4)
Cruciferous vegetables (g/d)	7.1 (20.0)	9.1 (22.7)	15.6 (28.1)	6.7 (17.9)	6.7 (17.4)	7.4 (16.6)
Fruit and berries (g/d)	111.4 (106.6)	104.1 (83.2)	123.6 (98.1)	68.8 (82.7)	75.2 (101.1)	64.1 (77.2)
Citrus (g/d)	21.0 (41.3)	26.6 (42.7)	21.0 (41.9)	9.8 (27.5)	8.8 (31.0)	7.6 (18.6)
Berries(g/d)	12.9 (23.2)	12.3 (21.7)	14.6 (24.7)	8.7 (21.9)	8.0 (16.9)	5.1 (11.0)
SSB (g/d)	63.4 (125.6)	45.3 (89.4)	22.5 (54.9)	123.5 (198.7)	90.3 (142.8)	88.2 (145.0)
Low-calorie beverages (g/d)	48.6 (157.5)	35.6 (101.6)	27.2 (141.8)	44.0 (154.6)	60.5 (169.4)	44.2 (127.9)

	**1st SFFQ All women**	**1st SFFQ Subsample women**	**2nd SFFQ Subsample women**	**1st SFFQ All men**	**1st SFFQ Subsample men**	**2nd SFFQ Subsample men**
n	885	115	115	716	65	65
Low-fiber soft bread (slice/month)	12.9 (17.9)	15.7 (18.5)	14.9 (18.9)	18.3 (22.1)	19.3 (20.5)	16.9 (18.9)
High-fiber soft bread (slice/month)	11.3 (15.6)	15.7 (17.6)	14.9 (18.5)	14.8 (21.5)	20.9 (28.2)	18.8 (20.9)
High-fiber crispbread (slice/month)	8.8 (14.0)	13.0 (17.3)	12.0 (15.9)	6.8 (13.4)	9.1 (14.8)	7.4 (12.8)

Dietary data and Biomarkers

Fatty fish, (g/d)	17.6 (16.5)	18.0 (17.3)	19.4 (18.1)	20.9 (22.9)	20.9 (18.3)	19.8 (21.4)
Lean fish and shellfish (g/d)	15.3 (12.8)	15.8 (13.4)	16.3 (14.0)	18.8 (24.7)	20.1 (15.9)	17.3 (11.6)
Fish total (g/d)	34.7 (26.2)	35.2 (28.2)	36.5 (27.3)	42.1 (41.1)	44.1 (28.2)	38.8 (27.7)
Vegetables total (times/month)	25.8 (15.6)	26.5 (12.6)	26.7 814.7)	19.8 (12.6)	20.7 (9.9)	20.8 (10.9)
Legumes (times/month)	3.8 (5.3)	5.3 (5.4)	5.0 (4.8)	3.4 (5.0)	4.3 (4.2)	3.7 (3.5)
Green leafy vegetables (times/month)	7.7 (8.4)	7.5 (8.1)	7.5 (6.7)	5.8 (7.6)	6.2 (6.5)	6.2 (6.3)
Cruciferous vegetables (times/month)	4.9 (6.2)	7.0 (6.7)	7.2 (6.5)	4.1 (5.9)	5.5 (4.9)	5.1 (5.4)
Fruit and berries (times/month)	26.7 (22.3)	29.5 (19.2)	29.9 (18.7)	17.2 (16.8)	20.8 (17.1)	22.1 (15.5)
Citrus (times/month)	8.5 (10.2)	8.1 (8.8)	6.7 (8.3)	5.5 (6.7)	5.2 (5.6)	4.7 (5.2)
Berries (times/month)	5.2 (7.5)	5.8 (7.1)	6.9 (7.8)	3.0 (5.6)	4.3 (6.4)	4.7 (6.3)
Sugar-sweetened beverages (times/month)	2.8 (6.8)	2.6 (6.1)	2.1 (6.1)	5.0 (8.7)	5.9 (9.2)	4.6 (6.5)
Low-calorie beverages (times/month)	3.0 (8.1)	2.1 (6.7)	2.9 (7.8)	2.8 (7.2)	4.1 (9.4)	5.5 (12.9)

Supplementary Table 4. *Baseline daily nutrient intakes from the 4-d food record (4DFR) in the Malmö Offspring Study, and food intakes reported by the 4DFR that were not asked for in the short food frequency questionnaire (SFFQ) (n=1601)*

	Women<br>*n*=885	Men<br>*n*=716
Energy (kcal)	1790.8	2274.7
Carbohydrates (non fiber) (E%)	43.4	43.1
Fat (E%)	37.6	37.0
Saturated fat (E%)	14.3	14.0
Monounsaturated fat (E%)	14.2	14.1
Polyunsaturated fat (E%)	6.1	6.0
Protein (E%)	17.1	18.2
Fiber (g/kcal)	10.4	9.2
Sucrose (E%)	8.6	8.3
Monosaccharides (g)	26.9	28.4
Disaccharides (g)	52.8	62.6
Vitamin C (mg)	81.5	81.5
Folate (μg)	233.9	252.6
Retinol (μg)	470.7	554.8
β-carotene (μg)	1880.3	1891.8
Vitamin D (μg)	5.5	6.8
Vitamin E (mg)	12.5	13.5
Alcohol (g)	10.2	18.0
Iron (mg)	9.3	11.4
Zink (mg)	9.3	12.4
Magnesium (mg)	300.1	360.9
Calcium (mg)	800.0	911.7
Sodium (mg)	2727.8	3636.0
Total water (in beverages and food moisture)(g)	2157.4	2353.6
Whole grain (g)	33.2	37.2
Root vegetables(g)	17.4	16.3
Potatoes (g)	224.4	364.7
Breakfast cereals/porridge (portions)	0.1	0.1
Rice, pasta and other grains (g)	106.4	149.0
Nuts/seeds (g)	6.6	5.2
Red meat, non processed (g)	40.9	77.2
Processed meat (g)	20.0	35.7
Total red meat (g)	60.9	112.9
Poultry (g)	24.1	36.0
Meat/milk/cheese replacement products (portions)	0.05	0.03
Egg (g)	23.9	25.9
Total dairy (portions)	3.6	4.0
Yoghurt/sour milk (g)	78.3	68.4
Milk, non fermented total (g)	148.1	181.9
Cheese (portions)	1.6	1.9
Butter based spreads (g)	4.9	5.3
Oil-based spreads (g)	3.9	4.5
Sweets/pastry/desserts (incl. ice cream, sauce) (g)	68.5	64.3
Jam, sugar and honey (portions)	0.5	0.5
Salty snacks (g)	4.8	8.1

Food replacement products (portions)	2.0	3.0
Juice (g)	48.7	74.8
Tea (g)	118.4	53.2
Coffee (g)	294.9	328.5
Water (tap and bottled) (g)	732.7	643.3

Supplementary Table 5. *Spearman correlations* between food intakes assessed by the 4-d food record (4DFR) (g/d) and the short food frequency questionnaire (SFFQ) (times/month and g/d for fish intake), in a subsample with repeated measurements 1.6 y later (n=180) in the Malmö Offspring Study*

**Dietary factor**	**ϱ Baseline measurements *n*=180**	**ϱ Second measurements *n*=180**	**ϱ Mean of 2x4DFR against mean of 2xSFFQ *n*=180**
Fruit and berries	0.60	0.59	0.68
Citrus	0.51	0.41	0.52
Berries	0.39	0.32	0.44
Vegetables total	0.39	0.31	0.43
Legumes	0.32	0.32	0.34
Green leafy vegetables	0.16	0.28	0.26
Cruciferous vegetables	0.29	0.36	0.43
High-fiber soft bread total	0.51	0.68	0.74
High-fiber crisp bread	0.62	0.54	0.66
Low-fiber soft bread	0.38	0.35	0.47
Fish total (including shellfish)	0.19	0.35	0.38
Fatty fish	0.16	0.32	0.28
Lean fish and shellfish	0.28	0.28	0.30
Sugar-sweetened beverages	0.49	0.49	0.58
Low-calorie beverages	0.51	0.53	0.62

*P < 0.01 for all correlations except fatty fish from baseline measurements.

Supplementary Table 6. *Agreement between quartiles of intakes from specific fiber sources from the first and repeated 4-d food record (4DFR) in the Malmö Offspring Study (n=180)*

	Women Cross-classification (%)				Men Cross-classification (%)				All
**Dietary intakes**	**Perfect agreement (same quartile)**	**Same or adjacent quartile**	**Gross misclassification (Opposite quartile)**	**K**	**Perfect agreement (same quartile)**	**Same or adjacent quartile**	**Gross misclassification (Opposite quartile)**	**K**	**K**
Fruit and berries, total	40.0	81.8	4.3	0.20	43.1	73.9	4.6	0.24	0.21
Vegetables total	47.9	80.8	3.5	0.30	30.7	72.4	4.6	0.08	0.22
High-fiber bread total	38.2	79.1	6.1	0.18	41.5	67.7	6.1	0.21	0.19
Breakfast cereals/porridge	46.0	80.8	3.5	0.26	43.1	70.8	9.3	0.21	0.24

Dietary data and Biomarkers

Liquid chromatography-mass spectrometry

Metabolites were profiled in EDTA plasma using liquid chromatography-mass spectrometry (LC-MS) with a UPLC-QTOF-MS System (Agilent Technologies 1290 LC, 6550 MS, Agilent Technologies, Santa Clara, CA, USA). Plasma samples stored at -80^{0}C were thawed on ice before metabolite extraction was performed by adding 120 μl extraction solvent (4^{0}C) to 20 μl plasma and incubating at 4^{0}C with mixing at 1250 rpm during one hour. After incubation the samples were centrifuged for 15 minutes at 14000 x g and the supernatants were transferred to glass vials. Extraction solvent consisted of 80:20 methanol/water (Liquid Chromatography grade). Samples were analyzed using two different LC-MS methods.

For the first method (HILIC Pos), plasma samples were separated on an Acquity UPLC BEH Amide column (1.7μm, 2.1 × 100 mm; Waters Corporation, Milford, MA, USA) maintained at 40 ^{0}C. Solvent A: H_2O with 10mM Ammonium Formate and 0.1 % Formic acid. Solvent B: Acetonitrile with 0.1% Formic Acid. Gradient: 0-3 min, 100-95 % B; 3-6 min, 95-80 % B; 6-13 min, 80-70 % B; 13-14 min, 70-40 % B; 14-16 min, 40% B; 16-17 min, 100 % B. The flow rate was 0.4 ml/min and the sample injection volume 2μl. The autosampler was kept at 16 ^{0}C. Mass spectrometry was performed in positive electrospray ionization. The sheath gas temperature was set at 350 ^{0}C and the sheath gas flow at 12 l/min. The drying gas flow was 14 l/min and was delivered at 200 ^{0}C. Mass spectra were acquired at a rate of 1 spectrum/s and the mass range was 70 -1000 m/z.

For the second method (C18 Neg), plasma samples were separated on an ACE C18 column (1.7 mm; 2.1 3 100 mm; Advanced Chromatography Technologies Ltd., Aberdeen, UK) using gradient elution (mobile phase A, water with 0.1% formic acid; mobile phase B, acetonitrile with 0.1% formic acid) with a flow rate of 0.3 mL/min. The gradient was as follows: 0 to 5 minutes, 0% to 15% B; 5 to 13 minutes, 15% to 95% B; 13 to 15 minutes, 95% B; 15 to 16 minutes, 95% to 0% B; 16 to 18 minutes, 0% B. Subsequent MS analysis was performed in negative ion mode with settings similar as was used in HILIC Pos.

Metabolite Identification

CMPF, proline betaine and β-carotene were identified using a synthetically produced standards (Toronto Research Chemicals, Toronto, Canada). The identity of the metabolites was confirmed by matching the plasma measurements of mass-over charge ratio and retention time with data acquired from the synthetic standards (Table S2).

Data Processing

Metabolite peak areas were integrated using Agilent Profinder B.06.00 (Agilent Technologies, Santa Clara, CA, USA). Quality-control samples were injected every 10 analytical samples, in order to ensure high analytical repeatability. Each metabolite was normalized using its own measurements in the quality-control samples. First, a low-order nonlinear locally estimated smoothing function was fitted to the metabolite signals in the quality-control samples as a function of the injection order. The a-parameter, reflecting the proportion of samples to be used when constructing the correction curve, was set to 2/3. Using this function, a correction curve for the analytical samples was interpolated, to which the metabolite measurements in the analytical samples were normalized [1]. The normalization was performed in R software (version 3.6.1; R Foundation for Statistical Computing, Vienna, Austria).

Reference:

1. Dunn, W.B., et al. Procedures for large-scale metabolic profiling of serum and plasma using gas chromatography and liquid chromatography coupled to mass spectrometry. *Nat Protoc* **2011**, *6*, p. 1060-83, DOI: 10.1038/nprot.2011.335.
